# “Be positive as well as realistic”: a qualitative description analysis of information gaps experienced by breastfeeding mothers

**DOI:** 10.1186/s13006-015-0036-7

**Published:** 2015-03-07

**Authors:** Marie Dietrich Leurer, Eunice Misskey

**Affiliations:** College of Nursing, University of Saskatchewan Regina Campus, #100 – 4400 4th Avenue, Regina, Saskatchewan S4T 0H8 Canada; Freelance Public Health Nutritionist (formerly with Regina Qu’Appelle Health Region and Saskatchewan Ministry of Health), 118 Patterson Drive, Regina, Saskatchewan S4S 3W9 Canada

**Keywords:** Breastfeeding, Perceptions, Information, Advice, Gap

## Abstract

**Background:**

Early breastfeeding cessation is common in many regions of the world despite high breastfeeding initiation rates and strong evidence of the health benefits to both mother and infant. This research investigated mothers’ perceived breastfeeding information needs in order to increase our understanding of this phenomenon.

**Methods:**

Surveys were distributed by public health nurses in a health region in Western Canada to mothers who had initiated breastfeeding and whose infants were six to eleven months old to learn more about their infant feeding experiences during the birth to six month period. Two open-ended survey questions asked: (1) What support or advice did you receive that helped you with feeding your baby?” and (2) “What support or information on feeding your baby would you have found helpful but did not receive?” A total of 191 eligible mothers submitted a survey (response rate 35%) between January and October 2012. Qualitative description was used in analyzing the responses of the women who provided comments for the open-ended questions.

**Results:**

While many mothers felt their information needs were met, others outlined important content areas where more information and guidance was desired. These areas included milk supply management, frequency/duration of feeds, proper latch and feeding positions, nipple care, expression/pumping, other nutrition sources, and realistic information regarding common breastfeeding concerns.

**Conclusions:**

The results suggest that several of the information gaps highlighted by respondents in this study closely align with commonly cited reasons for breastfeeding cessation including perceived insufficient milk supply, latching difficulties and nipple discomfort. The findings emphasize the need for lactation support with systematic measures to ensure breastfeeding mothers are universally provided with information on these key content areas. Lactation supports should reinforce critical information and seek feedback to ascertain that mothers have clearly understood the information provided. Ensuring mothers receive and understand key breastfeeding information is a modifiable factor in efforts to increase breastfeeding duration rates.

## Background

The infant and maternal health benefits that accrue from breastfeeding are well documented in the literature [1,2]. Despite the World Health Organization’s recommendation that infants should be exclusively breastfed for the first six months of life for optimal health, global infant feeding practices fall well short of this goal. Breastfeeding initiation rates have continued to increase globally but decline dramatically by the time infants are six months of age. Even in higher income countries with considerable lactation support services, the rates of exclusive breastfeeding for six months remain low. In Canada, 89% of new mothers initiated breastfeeding in 2011–2012, but only 26% exclusively breastfed for six months [3]. Similarly, initiation rates were 76.5% in the United States, 81% in the United Kingdom and 96% in Australia (for infants born in 2010) with exclusive breastfeeding rates for six months of 17%, 1% and 15% respectively [4-6]. Significant health care savings could be realized if breastfeeding duration rates could be extended. Bartick and Reinhold estimated that if 90% of mothers in the United States breastfed exclusively for six months, 911 deaths would be prevented and 13 billion USD saved annually [7]. Similarly, a review of research using quantitative economic modelling in the United Kingdom estimated significant potential savings with achievable increases in breastfeeding including over 17 million GBP saved annually from costs to treat four acute infant diseases and over 31 million GBP incremental savings from maternal health conditions over the lifetime of each annual cohort of first time mothers [8].

Considerable research has been conducted in an attempt to uncover the reasons why so many mothers initiate breastfeeding but add supplementary feedings or discontinue breastfeeding prior to six months [9-13]. Up to 60% of mothers who discontinue breastfeeding do so earlier than they had desired [10]. The most commonly cited reasons for early cessation include a perception of insufficient milk supply, maternal discomfort (particularly sore nipples), difficulty with breastfeeding techniques, maternal psychosocial factors, and return to work/school [9,14-16].

A variety of lactation supports, including both peer and professional as well as institutional and community based supports, have been shown to improve breastfeeding rates [17-20]. A randomized clinical trial found that community health nurse and peer counselor support increased the duration of breastfeeding in low-income mothers [21]. A Cochrane Review of supports for breastfeeding mothers found all forms of extra support (both professional and lay support) effectively increased the duration of breastfeeding, particularly the duration of exclusive breastfeeding [22]. However, to be effective such lactation supports need to be accessible and meet the needs of breastfeeding mothers.

Comprehensive lactation support includes ensuring that new mothers receive adequate, relevant information in a timely manner. A meta-synthesis of research examining mothers’ perceptions and experiences of breastfeeding support found mothers sought realistic, “accurate and sufficiently detailed information” and “practical help” ([23], p. 53). Similarly Graffy and Taylor found breastfeeding mothers want information about what to expect, practical help, and effective advice and suggestions [24]. Research assessing mothers’ versus clinicians’ perspectives of breastfeeding counselling uncovered communication gaps with clinicians believing they had provided information on breastfeeding duration and mothers’ return to work more often than mothers reported receiving such information [25].

In an effort to gather experiential wisdom, this study explored the perceptions of mothers who had initiated breastfeeding by asking them to identify both the helpful advice they had received and the information they did not receive but would have found helpful.

## Methods

### Data collection

This research was conducted within a health region in Western Canada. Purposive sampling was used with surveys distributed by public health nurses (PHNs) during attendance at child health clinics (CHC) where most infants receive their routine immunization. The survey was developed by both authors and was piloted with PHNs distributing 30 pretests of the survey at CHCs. The process was a participating pretest as respondents were notified that they were helping with the survey design through a cover letter describing the process. After filling in the survey, pretest respondents were asked for their feedback regarding whether any survey questions were confusing and whether they would recommend any additional questions be included. Nine respondents completed the pretest with all providing positive feedback regarding the survey questions, thus no modifications were made.

The survey contained a combination of 26 categorical and open-ended questions and was estimated to take about 15 to 20 minutes to complete unless the respondents chose to provide more detailed answers to the open-ended questions. There were no restrictions placed on the length of the responses to open-ended questions. Categorical questions provided information regarding the demographic characteristics of the survey sample and infant feeding practices from birth to six months such as the extent to which breast milk was offered at different time periods, what and when solids or other liquids were introduced and, whether vitamin D was offered. Other questions explored what influenced mothers to breastfeed, any major problems encountered and reasons for discontinuing breastfeeding if applicable. Mothers’ perceptions and feelings about their breastfeeding experience and their recommendations to improve breastfeeding support were explored in depth. Findings from those questions are reported elsewhere [26]. This paper focuses on mothers’ perceived information needs and their responses to two specific open-ended questions: 1) “What support or advice did you receive that helped you with feeding your baby?” and 2) “What support or information on feeding your baby would you have found helpful but did not receive?”

The recruitment process involved the PHNs first determining the eligibility of attendees at CHC based on the infant record and then offering the survey envelope (containing a one page description of the research, a consent form, the survey and a stamped envelope addressed to the lead author) to eligible mothers along with a brief oral description of the survey and its purpose. Mothers could also help themselves to a survey envelope from recruitment poster displays in the public health office waiting room. Surveys were distributed to eligible mothers at all CHC sites in the health region (three urban and five rural). Mothers were not given time to complete the survey at CHC but rather were asked to take the envelope home. They were given the option to complete the paper version of the survey or an electronic version. Those who completed the paper version signed the consent form and returned it in the mail with their survey. Those who completed the survey electronically were presented with an online consent form indicating their consent was implied when they clicked on the “begin survey” button. Quotes of comments reported in the findings are designated either E (electronic online survey) or M (mail-in survey). Ethical approval for this research was received from both the University of Saskatchewan Behavioural Research Ethics Board (BEH 11–278) and the Regina Qu’Appelle Health Region Research Ethics Board (REB 11–74).

Eligibility criteria for survey respondents required that the mother had initiated breastfeeding and that their infant was between six months and one day less than twelve months of age during the study period. This age range allowed for exploration of infant feeding practices and experiences during the first six months while attempting to reduce recall bias by soliciting relevant information close in time to the period of interest. Infants in this health region routinely receive their third immunization during a visit to a PHN at a CHC at six months of age. Surveys were distributed to 551 eligible mothers from January to July 2012. In total 191 mothers completed the survey (response rate 35%) with 138 mailing in the survey and 53 choosing to complete the online option with the electronic survey hosted by the health region. The response rate was consistent with the rates for similar internet and mail surveys [27,28].

### Data analysis

All responses were analyzed using either quantitative or qualitative analysis. Qualitative description, a method useful in exploring the experiences of health care system consumers, was used to analyze responses to the open-ended questions in the survey that are the focus of this paper [29-31]. Initial analysis was conducted with the assistance of QDA Miner® qualitative software by the lead author, a nurse researcher with expertise in qualitative analysis who worked for many years providing breastfeeding support as a PHN. All narrative responses for each open-ended question were placed into categories with inductive reasoning guiding the search for commonalities and emerging patterns. Categories were developed and then merged or collapsed through this constant comparative analysis, typical of qualitative research [32,33]. The second author, a public health nutritionist specialized in infant and early childhood nutrition, reviewed the categorization of each narrative response and provided feedback regarding the naming of categories and placement of specific responses before agreement on the final categorization was reached.

## Results

Table 1 outlines the demographic characteristics of survey respondents. Respondents were almost evenly split between primiparous and multiparous, and were most commonly 26 to 35 years of age with infants six to seven months of age. Respondents tended to be relatively well educated and had a higher household income as compared to the general provincial population [34]. About 85% of respondents had a postsecondary degree, diploma or certificate. While the majority resided in the city, the 33% of rural respondents in this study sample are overrepresented, since 24% of the health region population were rural residents when the study was conducted. Some breastfeeding support services may be less accessible to mothers in rural areas of the region.

**Table 1:** **Demographic characteristics of respondents**

**Characteristic (N = 191)**	**Frequency**	**Percentage**
*Parity*		
Primiparous	94	49.2
Multiparous	97	50.8
*Gestation at birth (missing = 4)*		
Term	178	93.2
<37 weeks	9	4.7
*Single/multiple birth*		
Single birth	189	99.0
Multiple birth	2	1.0
*Maternal age in years (missing = 1)*		
12-18	1	0.5
19-25	18	9.4
26-30	84	44.0
31-35	64	33.5
36-40	23	12.0
40+	0	0
*Education*		
Some high school	1	.5
High school diploma	12	6.3
Some post-secondary	14	7.3
Post-secondary qualification	63	33.0
University degree	101	52.9
*Household Income (Canadian $) (missing = 25)*		
<20,000	5	2.6
20,000-49,000	12	6.3
50,000-79,000	28	14.7
80,000-109,000	51	26.7
>110,000	70	36.6
*Employment status (missing = 1)*		
Not employed	24	12.6
Back at employment	13	6.8
On maternity leave	148	77.5
Other	5	2.6
*Home location*		
City	132	69.1
Town/village	44	23.0
Farm/acreage	15	7.9
*Aboriginal status (missing = 1)*		
No	185	96.9
Yes	5	2.6
*Age of infant (missing = 1)*		
6 months	92	48.2
7 months	66	34.6
8 months	15	7.9
9 months	4	2.1
10 months	4	2.1
11 months	3	1.6
12 months	6	3.1

Written responses ranged widely from a few words to longer quite detailed answers. With regard to the two open-ended questions examined here, mothers shared their perceptions of the support/advice they had found helpful and the support/information they would have found helpful but did not receive. The vast majority of mothers reported they had received some form of helpful support/advice. Only 4% of those who completed the first question felt they had not received any helpful support/advice. Given the open-ended, non-categorical nature of the question, mothers identified the sources and/or content of the information they had received. Respondents often identified multiple sources of helpful information with the most common being nurses (41%), family (20%), written material (15%), friends/other moms (13%), previous experience breastfeeding (12%), lactation consultations (11%), physicians/nurse practitioners (7%), pre or postnatal group education classes (7%) and the internet (7%).

Hospital staff (mother/baby unit) assisted in trying to feed right after birth. I had called public health for advice regarding my baby being fussy and was given some good advice. The public health nurse also came for a home visit to weigh my baby as I was concerned he was not eating enough. (M75, primip, stopped breastfeeding at 6 months)

My mom and my wisdom I had from my first born. There is a lactation consultant, she was very helpful. (M84, multip, continuing to breastfeed at time of survey)

From my doctor I received good advice explaining what foods I should and shouldn’t give to baby. Nurse at hospital gave pamphlets showing and teaching me to do the proper nutrition from the start and through the different stages of baby’s development. It helped me a lot because it was very informative and knowledgeable. (M49, primip, continuing to breastfeed at time of survey)

Books and pamphlets from health region, public health nurse, friends, family, internet. (M41, multip, continuing to breastfeed at time of survey)

Those mothers who indicated they had received specific helpful information most commonly welcomed advice regarding latch and feeding positions, frequency and duration of feeds, what to feed baby, milk supply, pumping, and nipple care.

Hospital nurses - proper latching, how often and how long I should feed, changing sides. (M103, primip, continuing to breastfeed at time of survey)

The health nurses were very helpful, coming back to the house twice to assist with latching. Also at 4 months, my milk supply decreased, and the health nurses were again very helpful, providing information on pumping and herbs, etc. (E23, multip, continuing to breastfeed at time of survey)

The public health nurse that visited once I was home was extremely helpful. She helped with my latch and position and things got easier after that. (M68, primip, continuing to breastfeed at time of survey)

Make sure they’re latched on properly- in beginning put nipple cream on after each feeding. Be patient, relax. If you have a bad experience with nipple cracking at one feeding the next one can only be better. (M99, primip, stopped breastfeeding at 6 months)

While it is reassuring that many mothers felt they had received helpful support and advice from a variety of sources, this paper emphasizes the information deficits reported by many mothers revealing inconsistencies in the level of breastfeeding support and a lack of individualized services in some instances. Most mothers were able to identify information they would have found helpful but did not receive with only 18% of those completing the second question reporting they felt they had received all the support and information they needed.

Below are the main categories identified from answers to the question “What support or information on feeding your baby would you have found helpful but did not receive?”

### Breastfeeding frequency/duration

A common information gap mentioned by respondents was a lack of clarity regarding feeding frequency and length. It was evident that new mothers struggled to determine how often to breastfeed their baby and for how long, particularly in the early days. Their comments expressed a desire for more guidance in learning to read their baby’s cues and for reassurance that their baby was receiving sufficient milk with demand feeding.

The only thing I would have liked to know was how different types of babies eat. Some take forever, some just feed for comfort because I was feeding for too long on each side and that’s why I was in so much pain until the doctor told me no longer than 15 minutes each side each feeding. (M106, primip, stopped breastfeeding at 5 months)

Demand feeding, knowing when you’re feeding your baby too much. (M122, primip, continuing to breastfeed at time of survey)

Someone to explain how often a baby eats when breastfed. It would have better prepared me for only getting an hour or two of sleep and definitely would have helped with my coping of postpartum depression if I knew what to expect. (E7, primip, stopped breastfeeding at 2 months)

### Management of milk supply

Respondents felt uninformed regarding the management of their milk supply. A common concern was uncertainty regarding how to adapt their milk supply to their baby’s needs and how to assess the adequacy of their current supply. Some mothers wished they had received more clear guidance on how to maintain or increase their milk supply. Others alluded to differing health care provider (HCP) advice such as divergent opinions on the off-label use of domperidone as a galactagogue. A systematic review and meta-analysis concluded domperidone may be effective in increasing milk production, however the authors recommended non-pharmacological approaches, including lactation education, are preferred given the potential for adverse side-effects and the need for further research [35].

It would be good to let mommas know that their milk may not come in right away and that it’s nothing to worry about. (M81, primip, continuing to breastfeed at time of survey)

I would have loved to know how to keep your milk supply up when your body is not creating enough. This happened to me for a few weeks before my milk dried up. (E2, primip, stopped breastfeeding at 4 months)

There seems to be a gap between obstetricians and family doctors after/around six weeks. Milk supply decreased with both kids once birth control started. Domperidone worked and stopped birth control pills, but had to initiate by requesting from obstetrician. (M77, multip, continuing to breastfeed at time of survey)

In contrast, other mothers wanted more advice on dealing with engorgement, strong let down, and issues related to “too much milk”.

Prepare (mothers) for the engorgement phase. I didn’t realize what was really going on and that it would only be like 24 hours. (E30, multip, continuing to breastfeed at time of survey)

I would have started pumping earlier on as I had so much milk. I was choking my baby all the time because it was coming so fast. This was more me learning my baby. (M45, primip, continuing to breastfeed at time of survey)

I would have liked more information about different types of milk and too much milk since this was an issue. (M101, multip, continuing to breastfeed at time of survey)

Some responses hinted at a lack of confidence in their body’s ability to provide sufficient nourishment with mothers seeking reassurance that their baby was receiving enough “ounces”.

Maybe some more info on how many ounces of milk that a certain weight baby will eat. Helpful for when pumping and feeding from the bottle. (M103, primip, continuing to breastfeed at time of survey)

My one breast produced a lot of milk while the other didn’t. I was confused about how to help with this, how much milk my baby got and how to get him to feed enough so that he didn’t get painful gas (when he got too much fore milk). (M63, multip, stopped breastfeeding at 6 months)

### Latch and feeding positions

Mothers sought practical advice on breastfeeding techniques including how to effectively latch their baby and different feeding positions they could use, a particular need in the early days. They described wanting practical help with someone not only showing them how to latch their baby, but also allowing them to practice such skills themselves in order to develop confidence in their own breastfeeding ability.

I wish the nursing staff would use a hands-on approach when showing you how to latch your baby followed by a long period of you showing them how to latch your baby. I had a lot of nurses latch my baby for me at the beginning and I wish they would have let me do it with their supervision so I didn’t feel unsure when I left the hospital. (M40, primip, continuing to breastfeed at time of survey)

In my unique case, I just wish I had understood positioning for latch and pumping better prior to having our baby. (M78, primip, stopped breastfeeding at 5 months)

I did not receive sufficient support or attention from the mother baby unit in the hospital. I would have found it helpful for someone to show me how to work the breast pump, different ways to hold my baby, and how to ensure he was latched properly. (M75, primip, stopped breastfeeding at 6 months)

Some mothers wished they had been given more advice about effective feeding positions and encouragement to use a variety of positions.

I was shown different ways or positions of nursing our baby, but not told to switch up positions regularly. As a result I had many plugged ducts. (M124, primip, continuing to breastfeed at time of survey)

### Expression/pumping

Some mothers wished they had been given more information on how to express or pump breast milk. While a few mentioned hand expression, it seemed that many new mothers had a breast pump and wanted help in learning how to use it. A few mothers mentioned they wanted to pump to relieve excess milk but more commonly they wanted to learn to pump so someone else could feed the baby either so they could leave the baby for a period of time or so that a partner or family member could be more involved in the caregiving.

I would have liked more information on pumping and storing breast milk so that we could continue to give her breast milk after I had stopped breastfeeding. (E9, primip, stopped breastfeeding at 6 months)

I honestly think that it does cause fathers to feel left out some. Pumping so they can feed helps. Maybe that could be mentioned to new moms. That it is normal for fathers to feel that way. (M119, primip, continuing to breastfeed at time of survey)

Advice on pumping and what kind of pump to purchase. They are also expensive and if it doesn’t work for you, waste of money. Pumping is time consuming but getting baby onto bottle also can help with sanity and dad’s participation. (M30, multip, continuing to breastfeed at time of survey)

### Nipple care

Consistent with reports of difficulties with latch and positioning, mothers commonly described sore nipples as an issue. In addition to wanting guidance on prevention and what is normal, they also sought specific advice on comfort measures in order to relieve the pain and promote healing for those with nipple discomfort.

They could teach breast protection, for example, like what to do for severe sore nipples. It’s best to plan for prevention. All the doctor told me was I guess you can’t breastfeed this baby and put it on formula. (M66, multip, continuing to breastfeed at time of survey)

An explanation of the pain I should expect would have been helpful. I was fine at first, but after a week, it hurt a lot before I got used to it. Lanolin was very helpful, but I didn’t know what to expect or what was normal and it was really painful after we arrived home. Showing how to unlatch is also helpful. (E19, multip, continuing to breastfeed at time of survey)

It would have been helpful to know if there was anything I could have done to better “prepare” my nipples before feeding. I sought out a lot of information on my own though and felt as prepared as I could be. I was very realistic and knew it might be challenging. (M68, primip, continuing to breastfeed at time of survey)

### Realistic information on common breastfeeding concerns

Survey respondents felt pregnant women should receive more realistic information on common breastfeeding challenges. Comments suggested that although expectant parents learn about the benefits of breastfeeding, new mothers are often surprised by how challenging it can be, by the time required, and that they may experience periods of discomfort. Their narratives were clear that preparatory information should not only promote the benefits and joys of breastfeeding, but also recognize the realities and challenges a new mother can expect.

None with this baby, however with my first child I wish I had known that it would hurt at first. You are told that it should never hurt to breastfeed, but it does at first. (M138, multip, continuing to breastfeed at time of survey)

They need to help with the pain level expectations and help mothers get over the initial frustration of getting the babies to latch and to unlatch. (E23, multip, continuing to breastfeed at time of survey)

How tough it can be. Minor issues such as biting, lack of sleep, feeding strikes, plugged milk ducts, etc. Everyone talks about the benefits but not how hard it is on mom. If she was given info on problems and the benefits of breastfeeding then I still think she would breastfeed but at least be prepared for setbacks. (E34, primip, continuing to breastfeed at time of survey)

While mothers felt more information should be provided outlining common challenges, they also highlighted the importance of reassuring new mothers that it will get easier.

As a new mom it helps to listen to other new moms who successfully breastfed their babies. It is important to recognize that it is not always an easy process, but with patience and determination and the right support tools it is achievable. It is easy to give up without guidance and support. (M89, multip, continuing to breastfeed at time of survey)

You can be doing everything right and it may still hurt. With my first baby it hurt a lot to nurse on my left side in the beginning. He was latched properly and my nipples were normal. It just hurt a lot when my milk let down. I just kept nursing him, powered through and after a week or two it didn’t hurt anymore. (M59, multip, continuing to breastfeed at time of survey)

### Other nutrition sources

Some mothers felt that breastfeeding information is emphasized to the exclusion of other nutrition sources, resulting in a lack of information for those who decide to supplement or wean their baby to formula.

The focus was always on breast milk. Since I was having problems producing enough milk, info on formula would have been helpful. (M108, primip, stopped breastfeeding at 3 months)

As a result of my breast reduction surgery, supplementing was essential. The lactation consultant would not consider this as an option (while in hospital). I wish the consultant would have been receptive to my particular situation. (M123, primip, stopped breastfeeding at 2 months)

More information about formula usage . . . Teach you about the different kinds of formula and how to mix and use them. (M8, primip, stopped breastfeeding at 2 months)

Others sought more information on how and when to introduce solid foods, weaning techniques and how to prepare for a return to work.

At six months I had no idea what to feed him. There were many different people wanting to give advice, but I didn’t know who to believe. It would have been great to have a place to go and talk about solids. (M115, multip, stopped breastfeeding at 5 months)

How to incorporate food into baby’s diet while breastfeeding. (M112, multip, continuing to breastfeed at time of survey)

How to transition out of breastfeeding before going back to work at one year. (M20, primip, continuing to breastfeed at time of survey)

## Discussion

Mothers in this research identified not only the helpful information they had received but also information they felt had not been provided but which they would have found helpful. There were similarities between the helpful information received by some women and not by others. Given that women commonly experience some challenges as part of their breastfeeding experience, it is critical that this content is conveyed and reinforced to all mothers so they are equipped to deal with issues should they arise. While this survey found 43% of women reported they had a major breastfeeding problem or issue, McCann, Baydar and Williams found 70% of women reported at least one breastfeeding problem within the first month with this percentage decreasing to 45% and 29% at three and five months respectively [36]. The desire expressed by mothers in this research for guidance on breastfeeding frequency and duration, milk supply, latch and feedings positions, and nipple care is consistent with other research showing these are common breastfeeding challenges [36,37].

According to a Cochrane review, all forms of breastfeeding support increase breastfeeding duration with the authors concluding all women should universally be offered such support with proactive support more effective than reactive (women initiated contact) [22]. There is a need to ensure universal delivery of information to all breastfeeding mothers, particularly the key content areas identified by mothers in this study. Specifically, information must include an understanding of the basics of milk production and milk supply management, practical guidance that facilitates development of breastfeeding skills related to latch and positioning, realistic expectations regarding the benefits and challenges of breastfeeding, instruction on hand expression and introduction of other nutritional sources, and individualized instruction on pumping techniques as required.

A cause for concern is the similarities between many of the information deficits reported in this research and frequently cited reasons for breastfeeding cessation. Perceived insufficient milk supply is the most common reason for breastfeeding cessation [9,13,15,38]. Mothers seek guidance regarding how to manage their milk supply, and related to this, issues of frequency and duration of feedings particularly in the early days. Li, Fein, Chen and Grummer-Strawn found difficulties with latch and milk supply were the main factors in breastfeeding cessation within the first two months [39]. Other information needs identified (latch and feeding position, and nipple discomfort/care) are also prominent in the literature as factors in early breastfeeding cessation [14,15]. Once breastfeeding is well established, the desire for more information on other nutrition sources may impact the low rates of exclusive breastfeeding for six months if early introduction of complementary foods is related to a lack of knowledge regarding current recommendations and the rationale behind them. Thus, HCPs need to tailor the timing of information provided to coincide with the breastfeeding stage and unique needs of each mother with a recognition that mothers have evolving information needs throughout their lactation experience.

Some of the quotes from mothers allude to inconsistencies in the advice received such as conflicting advice regarding length and frequency of feeds, and strategies to increase milk supply. This finding corroborates with other research reporting incorrect or conflicting breastfeeding advice being offered with mothers desiring accurate and consistent messages [23,40]. Montalto, Borg, Buttigieg-Said and Clemmer found incorrect advice from HCPs to be a major factor in early breastfeeding cessation [41]. Such reports suggest the need to ensure HCPs working with lactating women have sufficient evidence-based knowledge and training to provide effective breastfeeding support.

One information gap identified, that was not mentioned by mothers who felt their information needs were met, related to a desire for more realistic information about common breastfeeding challenges and how to overcome these. Mothers felt breastfeeding education and promotion should normalize common challenges in order to help mothers anticipate and adjust to these challenges without feeling like a failure. These perceptions are supported in other literature reporting a clash between breastfeeding ideals and the realities of breastfeeding experienced by mothers [42]. Williamson, Leeming, Lyttle and Johnson found there was a tension between women’s actual breastfeeding experiences and the message that breastfeeding is easy and natural [43]. The researchers recommended “greater awareness and understanding of breastfeeding difficulties so that breastfeeding women are less likely to interpret these as a personal shortcoming in a manner which disempowers them” ([43] p. 434). Kelleher found women who experienced pain and discomfort were often surprised and anxious about this occurrence, with the physical impact of these having a negative effect on their desire to continue breastfeeding [44]. Breastfeeding promotion efforts and counselling could normalize common challenges in order to help mothers anticipate issues and enhance confidence in their ability to overcome such challenges.

Dennis described breastfeeding self-efficacy as a mother’s confidence in her breastfeeding ability with self-efficacy determining the amount of effort, perseverance and resiliency a mother exhibits, particularly when encountering challenges [45]. Other research has supported self-efficacy as an important and modifiable influence on breastfeeding duration [46,47]. Several of the information gaps identified in this research provide direction to HCPs by describing opportunities consistent with confidence-enhancing strategies outlined by Dennis [45]. First, HCPs can reinforce and provide encouragement for breastfeeding successes seen in their clients. This could include observing mothers practicing skills such as latching baby properly in order to provide reassurance that they are developing the necessary breastfeeding skills and capabilities. Second, given the inconsistencies in some of the advice offered to mothers as indicated in the quotes from this research, HCPs should strive to provide consistent, evidence-based advice. Third, HCPs should provide anticipatory guidance that normalizes the typical breastfeeding experience including advice on common challenges and strategies to address these. Such anticipation and acknowledgement that mothers often have to overcome breastfeeding challenges would help reduce anxiety and promote critical thinking to search for solutions to problems as part of a normal breastfeeding experience. Finally, although this paper is focused on key content areas, the manner in which this information is provided also matters. HCPs should provide information in an encouraging and supportive manner that provides reassurance and builds confidence.

Mothers also identified a desire for more information about expression or pumping. According to research conducted in the USA, there is a trend toward increased prevalence of milk expression with 85% of breastfeeding mothers with infants 1.5 to 4.5 months of age having expressed milk, and approximately one quarter of mothers of infants 1.5 to 9.5 months regularly expressing milk [48]. Reasons commonly cited for expression include poor latch or sucking in the early postpartum period, mother’s comfort, to increase milk supply, and to obtain breast milk for someone else to feed, either to involve the father in feeding or to continue breastfeeding upon return to employment [25,48,49]. The findings of this research are consistent with other research showing mothers feel clinicians provide insufficient information on pumping, breast milk storage and maintenance of milk supply, particularly when resuming employment [25]. While more research is needed to assess the overall health benefits and challenges to mothers and infants related to this increased milk expression [49,50], it is clear that since many mothers are expressing breast milk there is a need to routinely assess for this practice and offer guidance on correct techniques, including hand expression, as part of breastfeeding counselling.

The finding that a significant number of mothers perceive their breastfeeding information needs are not being met is supported by previous research [25,51,52]. Such findings not only provide direction for HCPs who work at an individual level with breastfeeding mothers but also for those responsible for healthcare system level delivery of breastfeeding programs. To deliver effective breastfeeding support, HCPs not only must have the necessary training to provide consistent and evidence-based breastfeeding information, but must communicate with one another and follow established breastfeeding guidelines [52]. Healthcare delivery systems should ensure HCPs have the time and support necessary to receive adequate training and have networks that facilitate communication regarding implementation of breastfeeding guidelines. It should also be acknowledged that HCPs provide breastfeeding support within the context of other broad sociocultural factors that also impact a mother’s breastfeeding experience.

Breastfeeding support should be offered throughout the continuum from the prenatal period until weaning to all mothers with particular content emphasized at critical times. Researchers found mothers who received individual counselling prenatally were 55% less likely to cease breastfeeding before 6 month [53]. Similarly McLeod, Pullon and Cookson found shorter breastfeeding duration was associated with women who felt they received insufficient breastfeeding information during the prenatal period [51]. The first few weeks postnatal is a particularly critical time. Researchers found assistance breastfeeding within 30 minutes of birth had a positive impact on continued breastfeeding at 24 hours and two weeks [54]. Women who received instruction on positioning and latch in the immediate postpartum period were 30% less likely to stop breastfeeding before six months [53].

Continued support following hospital discharge until weaning should be included as part of a seamless program of support. Lewallen et al. found 35% of those who had initiated breastfeeding had stopped by eight weeks with insufficient milk cited as the most common reason [15], illustrating that the first two months is a critical time to provide and reinforce information related to milk production, responding to infant cues, and assessing infant growth. In some instances breastfeeding support following hospital discharge is not offered to all mothers [15]. Even beyond the first two months, women have ongoing information needs. Women in this research identified learning needs common after breastfeeding is well established including more information related to the introduction of other nutrition sources and expression/pumping. Taveras et al. found women felt they were not provided with sufficient information regarding how to continue breastfeeding when they returned to work [25].

Limitations of this research firstly relates to the data being drawn from a written survey and thus lacking the rich detail of other data collection formats which provide an opportunity to ask further probing and clarifying questions. Second, given the self-selected nature of this survey, the respondents’ views may not reflect those of all women in the region. For example, the respondents in this sample had higher income and education levels than the population of the region in which the study was conducted. The results therefore may not reflect the experiences of more diverse and/or disadvantaged populations, including the significant Aboriginal population in the health region, which was underrepresented. Finally, the responses are specific to the services as experienced by mothers of this particular health region and may not reflect the breadth of lactation support services in other areas.

Further research might explore what systemic strategies are successful in ensuring universal delivery of key breastfeeding information to all new mothers. It might also investigate the variety of information sources available to new mothers and their preferred communication channels for receiving critical information. Such enquiry may highlight the preferences of new mothers for face-to-face guidance versus less personal, but potentially more timely and accessible information sources, such as telephone hotlines and the internet. Finally, further exploration into the factors behind the increased prevalence of milk expression/pumping may provide guidance regarding the information needs of those mothers.

## Conclusions

The findings highlight the importance of breastfeeding support services delivering universal, high quality, consistent advice to all breastfeeding mothers with a focus on ensuring key content areas are explained and reinforced in order to confirm mothers’ information needs are met. While the information gaps mothers identified in this study may reflect the lactation services offered in this particular health region, it is clear mothers’ desired guidance on the basics of milk production, feeding frequency/duration, proper latch and feeding positions, nipple care, expression/pumping and the introduction of other sources of nutrition. In addition, mothers felt that normalizing common breastfeeding challenges will better prepare new mothers for their breastfeeding experience and enhance confidence when things are not going smoothly. Mothers who perceive they have not received adequate instruction on these key topics may be at risk for premature breastfeeding cessation. Universal instruction of key content areas is a modifiable factor in efforts to improve breastfeeding duration.
